# Optimal Conditions for Diapause Survival of *Aprostocetus fukutai*, an Egg Parasitoid for Biological Control of *Anoplophora chinensis*

**DOI:** 10.3390/insects12060535

**Published:** 2021-06-09

**Authors:** Xingeng Wang, Nathalie Ramualde, Ellen M. Aparicio, Matteo Maspero, Jian J. Duan, Lincoln Smith

**Affiliations:** 1 USDA-ARS Beneficial Insects Introduction Research Unit, Newark, DE 19713, USA; ellen.aparicio@usda.gov (E.M.A.); jian.duan@usda.gov (J.J.D.); 2 USDA-ARS European Biological Control Laboratory, 34980 Montferrier-sur-Lez, France; nramualde@ars-ebcl.org (N.R.); link.smith@usda.gov (L.S.); 3 Department of Food, Environmental and Nutritional Sciences, University of Milan, 20122 Milan, Italy; matteo.maspero@agrigeorgia.ge; 4 Ferrero Hazelnut Company, 2100 Chitatskari, Georgia; 5 USDA-ARS Invasive Species and Pollinator Health Research Unit, Albany, CA 94710, USA

**Keywords:** *Anoplophora*, *Aprostocetus*, classical biological control, egg parasitoid, diapause, invasive forest pest

## Abstract

**Simple Summary:**

Diapause is a critical state of an insect’s life cycle when it undergoes arrestment of growth and/or reproduction to survive adverse environmental conditions and/or food shortage. *Aprostocetus fukutai* is a specialist egg parasitoid of the citrus longhorned beetle, *Anoplophora chinensis*, a high-risk invasive woodboring pest. The parasitoid overwinters as diapausing mature larva in the host egg and emerges in early summer in synchrony with the egg-laying of *A. chinensis*. Here, we determined the optimal conditions for diapause survival of this parasitoid. We showed that the parasitoid had a low (36.7%) diapause survival rate inside host eggs laid on potted plants due to desiccation or tree wound defense response under semi-natural conditions. Under laboratory rearing conditions, when parasitized host eggs were extracted from wood, the parasitoid did not survive at low humidity (44% RH). Survival rate increased with humidity, reaching the highest at 100% RH. Survival rate also increased with increasing chilling period temperature from 2 to 12.5 °C. Post-diapause developmental time decreased with increased humidity or temperature, but the reproductive fitness of the parasitoid was not significantly affected by the temperature regimes. Overall, high humidity (100% RH) and mild temperatures (12.5 °C) are the most suitable survival conditions for the diapausing parasitoid and thus should be used in laboratory rearing.

**Abstract:**

*Aprostocetus fukutai* is a specialist egg parasitoid of the citrus longhorned beetle *Anoplophora chinensis*, a high-risk invasive pest of hardwood trees. The parasitoid overwinters as diapausing mature larvae within the host egg and emerges in early summer in synchrony with the egg-laying peak of *A. chinensis*. This study investigated the parasitoid’s diapause survival in parasitized host eggs that either remained in potted trees under semi-natural conditions in southern France or were removed from the wood and held at four different humidities (44, 75, 85–93 and 100% RH) at 11 °C or four different temperature regimes (2, 5, 10 and 12.5 °C) at 100% RH in the laboratory. The temperature regimes reflect overwintering temperatures across the parasitoid’s geographical distribution in its native range. Results show that the parasitoid resumed its development to the adult stage at normal rearing conditions (22 °C, 100% RH, 14L:10D) after 6- or 7-months cold chilling at both the semi-natural and laboratory conditions. It had a low survival rate (36.7%) on potted plants due to desiccation or tree wound defense response. No parasitoids survived at 44% RH, but survival rate increased with humidity, reaching the highest (93.7%) at 100% RH. Survival rate also increased from 21.0% at 2 °C to 82.8% at 12.5 °C. Post-diapause developmental time decreased with increased humidity or temperature. There was no difference in the lifetime fecundity of emerged females from 2 and 12.5 °C. These results suggest that 100% RH and 12.5 °C are the most suitable diapause conditions for laboratory rearing of this parasitoid.

## 1. Introduction

Diapause is a critical state of an insect’s life cycle when it undergoes arrestment of growth and/or reproduction to survive adverse environmental conditions and/or food shortage [1,2,3,4]. In insect parasitoids, many species have a long period of diapause that allows the parasitoid phenology to synchronize with that of their host [5,6]. Diapause may initiate at a predetermined point in an insect life cycle (i.e., obligatory) regardless of the environmental conditions or when particular stimuli occur that typically precede adverse conditions, such as short day-length (i.e., facultative). Regardless of the type of diapause, diapausing insects minimize energetic costs via metabolic depression during diapause to improve survivorship and fitness for post-diapause development [7,8]. Environmental conditions such as temperature and humidity can be critical for diapause survival as well as post-diapause development and reproductive fitness of parasitoids [8,9,10,11]. It is thus fundamentally important to understand the appropriate diapause conditions of biological control agents such as parasitoids in order to optimize biocontrol programs against insect pests [3,7,8,11].

The citrus longhorned beetle, *Anoplophora chinensis* Förster (Coleoptera: Cerambycidae) is a high-risk invasive hardwood tree pest [12]. The beetle is widely distributed in China, Korea and Japan, and occasionally found in Indonesia, Malaysia, the Philippines and Vietnam [12]. *Anoplophora chinensis* has been detected repeatedly in more than 11 European countries; although most of these invasive populations have been subsequently eradicated, they are still present in Italy and Croatia [13]. The beetle has also been intercepted frequently in Australasia and North America in live bonsai and nursery plants or occasionally in wood packaging material [12,14,15]. *Anoplophora chinensis* can attack a wide range of broadleaved trees in 36 families and over 100 plant species, including *Acer*, *Betula*, *Corylus*, *Populus* and *Salix,* as well as some orchard trees such as *Citrus* [12]. New introductions are an ongoing challenge. If invasive populations become permanently established outside of its native range, the beetle will cause significant ecological damage to forests and economic impact to forestry and citrus industry. Biological control, especially by means of self-perpetuating parasitoids, could be a viable option to reducing established and incipient populations in urban and natural forests, where intensive management methods such as chemical control and destruction of infested trees may be environmentally undesirable [16,17]. Furthermore, most of the beetle’s life cycle is spent in immature stages within the wood, where insecticide sprays are not effective unless applied as systemic treatments. Having effective parasitoids already available would allow us to rapidly develop a sustainable management tool for this invasive pest when eradication becomes untenable.

Wang et al. [18] recently reviewed parasitoid guilds associated with cerambycid woodborers, and characterized their life-history traits and macroecological patterns of host use. These authors found that the parasitoid guilds shift from more host specialist endoparasitoids to more generalist ectoparasitoids following the hosts’ ontogeny and the increasing inaccessibility of the host’s location. To date, *Aprostocetus fukutai* (Miwa et Sonan) (Hymenoptera: Eulophidae) is the only known egg parasitoid of *A. chinensis* from its native range, while other parasitoids commonly associated with this beetle in Asia are mainly larval ectoparasitoids, such as *Dastarcus helophoroides* (Fairmaire) (Coleoptera: Bothrideridae) and *Scleroderma guani* (Hymenoptera: Bethylidae), which are generalist species not recommended for biological control introduction due to risk to nontarget species [18,19]. Several native European hymenopteran parasitoids of woodboring beetles can parasitize *A. chinensis* larvae: *Spathius erythrocephalus* Wesmael, *Eurytoma melanoneura* Walker, *Calosota vernalis* Curtis, *Cleonymus brevis* Boucek, *Trigonoderus princeps* Westwood and *Sclerodermus* spp. [20]. In addition, a common North American braconid parasitoid species *Ontsira mellipes* Ashmead successfully attacked *A. chinensis* in a laboratory test [21,22]. These indigenous larval parasitoids may potentially help to control invasive woodborers via novel associations [23,24,25], but effective classical biological control would depend on the introduction of co-evolved host-specific parasitoids. Indeed, a recent example of successful biological control of exotic longhorned beetles involved the egg parasitoid, *Avetianella longoi* Siscaro (Hymenoptera: Encyrtidae) that has been successfully introduced from Australia into California to effectively suppress the invasive eucalyptus longhorned borer *Phoracantha semipunctata* (Fabricius) (Coleoptera: Cerambycidae) [26]. The same egg parasitoid has also been accidentally introduced into the Mediterranean region and successfully controlled *P. semipunctata* [17].

An adventive population of *A. fukutai* (initially identified as *Aprostocetus anoplophorae* n. sp. [27]) was found to parasitize *A. chinensis* eggs shortly after the detection of the beetle populations in 2002 in northern Italy [28]. The adventive population was likely introduced from Japan within parasitized *A. chinensis* eggs in bonsais [20]. In nature, *A. chinensis* larvae develop in 1–2 years (depending on the local temperature), pupation occurs in the early spring, adults emerge in later spring and early summer, and oviposition occurs from June to August [12]. *Aprostocetus fukutai* is native to east Asia (Japan and China) and appears to be widely distributed in China [27,29,30]. The parasitoid overwinters as mature larvae within the host’s eggshell, and as many as two or three generations are reported from North to South China [30,31]. In northern Italy, *A. fukutai* appears to have 1–2 generations: the first generation emerges in early summer in synchrony with the egg-laying peak of *A. chinensis,* and part of the progeny of the first generation develops to adults in late August to early September of the same year while the remainder are in diapause until the following summer [32]. The ability of overwintering parasitoid larvae to survive inside the host eggshell for up to 10 months is probably the most important factor responsible for its accidental introduction into northern Italy on live bonsai trees [20].

*Aprostocetus fukutai* is a gregarious parasitoid, producing about 10 offspring (≈85% females) per parasitized host (Wang et al., unpublished data). As high as 72% parasitism of *A. chinensis* was observed during the first few years of its detection in Northern Italy, and field observations did not find *A. fukutai* attacking other cerambycid species that are sympatric to *A. chinensis*, suggesting a high level of host specificity of the parasitoid [18,32]. High levels of parasitism of *A. chinensis* (80%) by *A. fukutai* was also observed in an abandoned orchard of pomelos in China [32]. However, information is generally lacking on the biology and ecology of *A. fukutai.* There is no reported study on the parasitoid’s diapause biology, which is critical for efficient rearing of this parasitoid for laboratory studies or field release against *A. chinensis*. Therefore, this study first measured diapause survival of this parasitoid in host eggs naturally laid on potted young trees under naturally fluctuating climatic conditions in an outdoor quarantine cage, and then determined optimal humidity and temperature conditions for its diapause survival in host eggs extracted from wood and held in containers in the laboratory.

## 2. Materials and Methods

### 2.1. Insects and Plants

All insect rearing and laboratory bioassays were conducted at the quarantine facilities of the USDA-ARS European Biological Control Laboratory (EBCL) in Montferrier-sur-Lez, France, and the USDA-ARS Beneficial Insects Introduction Research Unit (BIIRU) in Newark, DE, USA. At EBCL, laboratory colonies of *A. chinensis* were established initially using larval beetles collected in China in 2012 and in northern Italy (Lombardy) in 2002, respectively. Thereafter, the Italian colony was augmented each year with adults emerged from collections of cut sections of infested trees in the same areas in Lombardy. Both colonies were used to provide host eggs in this study. The colonies were maintained under the controlled room conditions (21–22 °C, 50–60% RH, 14L:10D). Adult beetles were fed with fresh maple (*Acer negundo*) or willow (*Salix alba*) twigs (depending on the availability) for a minimum of 10 days (maturation feeding) in 1 L glass jars prior to mating, and then paired for mating [33]. Mated females were then provided with small freshly cut *Salix* logs (2–3 cm diameter, 10–15 cm long) for oviposition. Eggs hatched in about 10 days. Young larvae were extracted from the logs and transferred to containers for rearing individually on a cellulose-based artificial diet [34]. After 5–6 months of rearing, mature larvae were put in a cold room (10 °C, dark) for a 3-month cold chilling, and then were returned to the controlled room conditions for pupation and adult emergence. A laboratory colony of *A. chinensis* was also established at BIIRU, using 50 larvae (from the Italian colony) provided by EBCL in May 2018 and maintained under the controlled room conditions (23 ± 1.5 °C, 45–60% RH, 16L:8D) using similar methods as those described above, except that only maple (*Acer* spp.) twigs and branches were used as adult food and oviposition medium for the beetle.

Parasitoids used in this study were collected as diapausing larvae after overwintering in parasitized *A. chinensis* eggs in April 2018 at two locations (45.53806 N, 8.98472 E; 45.55917 N, 9.00139 E) near Milan, Italy, with a permit issued by the Italian Plant Protection Services. Four *A. chinensis*-infested trees (*Acer saccharinum, Rosa* sp., *Platanus* sp. and *Malus* sp.) were cut down as a part of eradication treatments by the Regional Plant Health Service. Since adult *A. chinensis* females typically lay eggs on the lower trunk of trees [12], infested parts of the lower trunks of each tree were cut into sections (15–20 cm diameter, 10–15 cm length). A total of 19 sections cut from the four infested plants were temporally stored at a nearby secure field site (45.55611N, 8.98889E) under natural conditions until May, when they were transferred to EBCL quarantine and stored individually in sleeve cages in a walking-in growth chamber (20–21 °C, 70–90% RH, 14L:10D) for the emergence of adult parasitoids or beetles. In total, 23 female and 2 male *A. fukutai* emerged from three parasitized eggs in *A. saccharinum* and *Platanus* sp. portions, from 18 to 26 June 2018.

**Figure 1:**
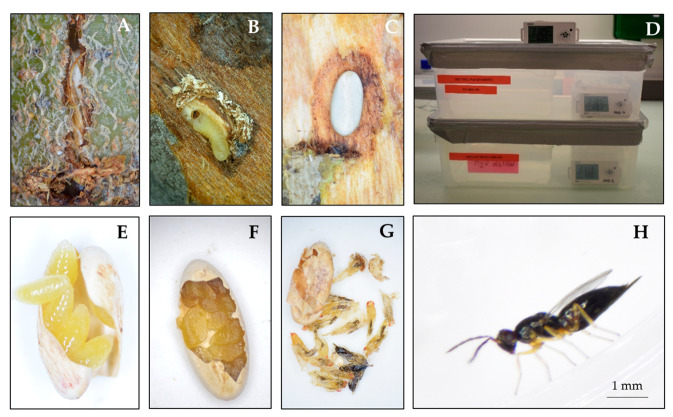
Laboratory rearing of the parasitoid *A. fukutai*: (**A**) T-shaped oviposition scar of *A. chinensis* in a *Salix* log containing an egg which was exposed to the parasitoid for parasitization, (**B**) an unparasitized egg (hatched with sawdust from larval feeding), (**C**) a parasitized egg (swollen) containing mature parasitoid larvae, (**D**) excised parasitized eggs placed in 24-well cell culture plates inside a humidity box, (**E**) heathy parasitoid larvae, (**F**) dead parasitoid larvae after diapause, (**G**) dead parasitoid pupae and unemerged adults after diapause, and (**H**) an emerged adult female parasitoid.

To collect host eggs for the rearing of the parasitoid, 2–3 freshly cut *Salix* logs (2–5 cm diameter, 10–15 cm long) were exposed to a mating pair of adult beetles in 1 L glass jars for 2–3 days which resulted in 2–3 host eggs per bough. Female beetles chew a small slit (3–4 mm long, transversally to the axis of the bough) in the bark and then oviposit mainly a single egg (perpendicularly to the slit). The pressure of the ovipositor during insertion often results in the upper layer of bark cracking to form a typical “T-shaped crack” or oviposition scar, with the egg partially visible (Figure 1A). The infested logs containing 3–5 freshly laid host eggs were then exposed to a mated female *A. fukutai* for 3–5 days, with 100% honey and water via cotton in a glass vial provided as food for the parasitoid. Exposed host logs were held for up to 15 days and then dissected to carefully extract the host eggs. Unparasitized eggs usually had hatched at the time of dissection, and the feeding activity of the young beetle larvae resulted in the presence of sawdust (Figure 1B), whereas parasitized eggs were swollen with the development of parasitoid larvae (Figure 1C). To reduce fungal infection, each parasitized egg was briefly dipped (10 s) in 5% bleach solution, rinsed in distilled water and then air-dried on a paper towel [34]. The parasitized host eggs were then placed individually in 24-well cell culture plates placed in humidity boxes (made of a polypropylene plastic box 32 × 18 ×10.5 cm with the lid sealed with duct tape) (100% RH, controlled by distilled water) (Figure 1D) under the controlled room conditions as described above. Part of the progeny of the first generation (<20%) could develop into the summer generation and emerged from mid-August to late September while the rest were in diapause. The summer generation was used to produce more parasitized host eggs. The parasitized eggs were held under the controlled room conditions for at least one month to allow the parasitoids to develop into mature larvae. By mid-October, the diapausing parasitoid larvae from the first and summer generations were moved to the diapausing conditions (10 °C, 100% RH and 24 h dark in a refrigerator) until May of the following year, when they were returned to the controlled room conditions for adult emergence.

### 2.2. Diapause Survival on Potted Trees under Semi-Natural Conditions

**Figure 2:**
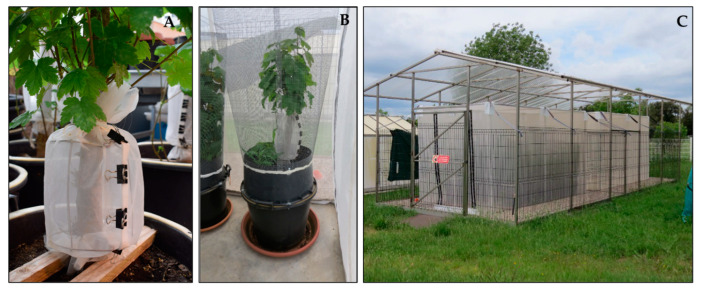
Exposure of *A. fukutai* adults to *A. chinensis* eggs on potted maple trees and rearing of the parasitoid under semi-natural conditions: (**A**) *A. chinensis* egg-infested tree exposed to parasitoid oviposition inside a sleeve cage, (**B**) potted tree with a hardware mesh cage to prevent the escape of adult *A. chinensis*, and (**C**) field test tent made of fine mesh in the certified outdoor quarantine enclosure at EBCL, protected by a plastic roof and metal fence.

The trials were carried out in a field insect tent (2 × 4 × 2.3 m) covering with a 200 µm nylon mesh outside the EBCL quarantine facility inside a fenced enclosure that was certified as a containment structure to prevent escape of insects (Figure 2). Potted trees (7–10 cm diameter at collar level) of sycamore maple, *Acer pseudoplatanus* L., were purchased from a commercial nursery in November 2017. The tree trunks were cut at 1.5 m height so that they would fit in the quarantine field tent. The cut wounds were treated with a copper sulfate solution to reduce fungal infection. The trees were kept outdoors throughout the 2017 winter and 2018 spring, to allow new branches to grow near the collar area, and were used in the summer 2018. A few branches with leaves near the trunk were kept to purposely provide a shelter for the beetles or parasitoids. The trees were infested by *A. chinensis* eggs inside the quarantine greenhouse (21–24 °C, 50–60% RH, natural light). A fine mesh sleeve cage was installed around the collar area enclosing up to 50 cm above the soil level, and a pair of mating *A. chinensis* adults was then released into each cage until approximately 10 oviposition slits were observed (2–3 days) (Figure 2A). After each oviposition site was marked, one mated female *A. fukutai* was released into each cage with water provided via cotton in a vial and honey droplets streaked on the sleeve. After the parasitoid had died (death occurred mostly within 7 days), all beetle oviposition sites were examined (10–15 days later). Unparasitized host eggs (i.e., hatched larvae), evidenced by extruded sawdust (Figure 1B), were removed, to prevent them from possibly cannibalizing parasitized host eggs. This was also a safety precaution to prevent potential escape of this quarantine pest. Further safety measures were taken by enclosing each tree with a cylindrical cage made of fine hardware mesh (Figure 2B) before the plants were then transferred to the quarantine field tent in early November 2018 (Figure 2C). The trees were watered once every two weeks and returned to the quarantine greenhouse in mid-May 2019 and monitored for the emergence of *A. fukutai*. By the end of September 2019, all the oviposition slits were dissected to determine the fate of each host egg (empty egg with an exit hole of the parasitoids or dead eggs containing dead parasitoid larvae, pupae, or adults (Figure 1F,G). The number of eggs killed due to tree defensive responses, such as sap exudation or rapid growth of plant tissue by the tree in response to oviposition wounds, was also recorded. We used a total of 21 trees, and the first set of eight trees were exposed to the female wasps that emerged in June 2018 from the field collection, and a second set of 13 trees were exposed to the parasitoids that emerged in August and September 2018.

### 2.3. Optimal Humidity

Parasitized eggs for this bioassay were prepared using similar procedures as described above for the rearing of the parasitoid. Host eggs were excised from the bark 10–15 days following their exposure to the parasitoids, or as soon as any unparasitized host eggs had hatched (based on extruded sawdust). All parasitized eggs were surface sterilized and individually placed in 24-well cell culture plates inside tightly closed humidity boxes (Figure 1D). There were four different humidity treatments (44, 75, 85–93 and 100% RH). The different levels of humidity were accurately controlled by using various saturated salt solutions [35] or distilled water for the 100% RH treatment (Table 1). Treatments for the 44, 75 and 100% RH used parasitized eggs from the first generation, while those for the 85–93% RH treatment used parasitized eggs obtained from the second generation in 2018. The humidity boxes were held under normal rearing conditions (22 °C 14L:10D) until mid-October 2018, when they were moved into an incubator under the conditions (15 °C, 12L:12D) for one month and then changed to conditions (11 °C, 10L:14D) for a 6-month chilling period. All parasitized eggs were checked immediately prior to the cold treatment, and those obviously dead were removed and dissected to determine survival before the start of chilling period. Following the chilling period, the humidity boxes were returned to the room rearing conditions and monitored daily for the emergence of wasps. After parasitoid emergence ceased in about 1–2 months, all ‘unemerged’ (presumably dead) eggs were rehydrated in water for 1 day and then dissected under a microscope to determine the developmental stage at death and the presence or absence of recognizable parasitoid larvae, pupae and pharate adults (Figure 1F,G). Our preliminary observations found that mortality of unparasitized host eggs was low (1–5%). Since parasitized host eggs had been held under the normal room rearing conditions for at least 1–2 months (i.e., parasitoids were allowed to develop into mature larvae), if final dissection did not find dead larvae, pupae or adults inside the eggshell, the parasitoids were considered to have died at the egg stage prior to cold chilling. It was also possible that some parasitoids might have died at larval stages at the onset of diapause. We thus measured the percentage of individuals emerged as adults from each parasitized egg, and considered this as a variable to measure the diapause survival of the parasitoid. The clutch size per parasitized host eggs was calculated based on the total number of emerged adults and dead larvae, pupae and adults from the dissection, while the percentage of female offspring was calculated by dividing the total number of adult females by the total number of adults from the rearing and dissection. Post-diapause developmental time was also calculated. The test initially consisted of 24, 54, 59 and 72 parasitized eggs for 44, 75, 85–93 and 100% RH, respectively (Table 1).

### 2.4. Optimal Temperature Regimes

Parasitized host eggs for this bioassay were also prepared using similar procedures as described above for the rearing of the parasitoid. To determine optimal temperature regimes for the diapause survival of *A. fukutai*, the parasitized host eggs were held at four different temperature regimes (2, 5, 10 and 12.5 °C). The distribution of *A. chinensis* in China is primarily in central and southern China [36]. Therefore, the tested temperature range reflects potentially different winter climatic conditions from central to southern China in the parasitoid’s native range. An additional treatment with the normal rearing conditions (22 °C, 14L:10D) was also added to determine if the parasitoid could complete development without a chilling period. Treatments of 5, 10 and 22 °C were conducted at EBCL, while treatments of 2 and 12.5 °C were conducted at BIIRU, using the same cohorts of parasitized eggs that were all obtained at EBCL. All parasitized hosts were obtained in September 2019 and held first under the normal rearing conditions (22 °C, 14L:10D) for about one month to allow the parasitoids to develop into mature larvae before they were set-up under the diapause conditions in the first week of October 2019.

For all treatments, the parasitized eggs were placed in 24-well cell culture plates in 100% RH humidity boxes (the most suitable humidity based on the above test) in corresponding temperature regimes in incubators (no light). Each treatment consisted of 25, 71, 83, 30 and 11 host eggs for the 2, 5, 10, 12.5 and 22 °C temperatures, respectively. After the end of the 6–7 months chilling period (Table 1), the humidity boxes were held at the room rearing conditions until the emergence of the adult parasitoids. All dead eggs were dissected as described above. During the dissections, a sub-sample of a total of 118 host eggs from different treatments were measured for length and width to determine the possible effect of host egg size on realized clutch size and offspring sex ratio of the parasitoid. The host egg volume was estimated using the formula (*V* = 4/3π ⋅ (*l*/2) ⋅ (*w*/2)^2^, where *V* is the volume of a prolate ellipsoid egg (Figure 1C) with length *l* and width *w*) [37].

To determine the possible effect of diapause temperature on post-diapause reproductive fitness, a sub-sample of 20 and 25 emerged females from 2 and 12.5 °C, respectively, were measured under the suitable conditions (23 ± 1.5 °C, 45–60% RH, 16L:8D) for their longevity and life-time fecundity by providing each female wasp with approximately 7 *A. chinensis* eggs per week in freshly-cut maple logs in a 1 L plastic jar until the female was dead. All exposed logs were dissected 15 days later when unparasitized host eggs had hatched to determine the number of host eggs parasitized.

### 2.5. Data Analysis

Percent survival of the parasitoid at different humidity or temperature regimes was analyzed using Generalized Linear Model (GLM) with a binomial distribution and a logit-link function to evaluate the effect of treatment and clutch size (considering potential mortality resulting from competition) as well as their interaction. Data on the proportion of female offspring were pooled from various replicates within each treatment and were also analyzed using GLM with a binomial distribution and a logit-link function to evaluate the possible effect of clutch size, diapause treatment and their interaction. Males and females of the same brood emerged almost during the same time, therefore both sexes were combined for the analyses of treatment effect on developmental time. Both developmental time and life-time fecundity were analyzed using one-way ANOVA. Female longevity data were subject to Survival Analysis (log-rank test) (no censored data). The relationship between host egg size and parasitoid clutch size was analyzed using linear regression. Prior to ANOVA, data were checked for normality. All analyses were performed using JMP^®^, Pro 14 (SAS Institute Inc., Cary, NC, USA, 1989–2019).

## 3. Results

### 3.1. Diapause Survival on Potted Trees under Semi-Natural Conditions

A mean ± SE of 10.2 ± 1.5 *A. chinensis* eggs were initially provided per potted tree. Of them, 30.4 ± 6.0% were parasitized. We observed sap exuded abundantly at some oviposition sites, and the oozing sap became quite solid as it dried outside the bark. Among unparasitized hosts, 16.2 ± 5.5% of them were dead at the egg or young larva stage due to desiccation, sap and/or fungal infection. In several cases, *A. chinensis* eggs or newly hatched larvae were drowned in sap or died stuck to sap. Among the parasitized host eggs, 60.0 ± 9.5% were dead due to desiccation and 22.8 ± 8.9% were expelled or crushed due to a defensive wound response of the tree after the diapause test in the field tent. Progeny emerged from the surviving host eggs post-diapause in June or July. There was no difference in the percentage of the progeny that successfully emerged from the first (35.7 ± 17.9%) and second (37.5 ± 18.3%) cohorts (*F*_1,13_ < 0.1, *p* = 0.984).

### 3.2. Optimal Humidity

**Figure 3:**
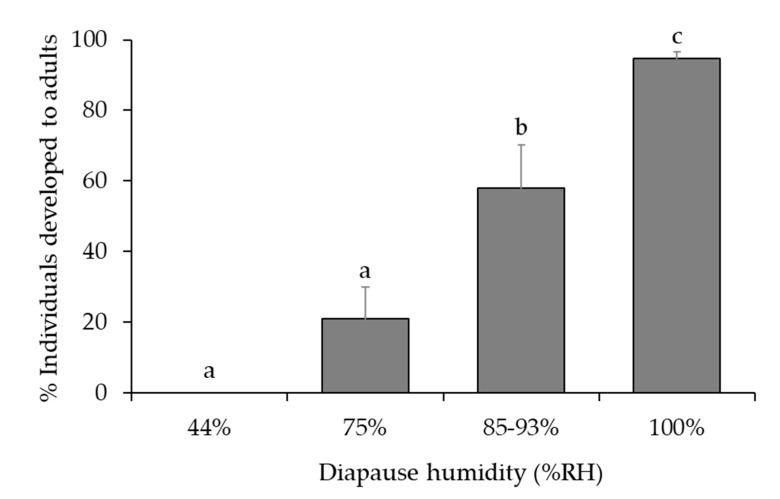
Effect of humidity conditions (see Table 1) on diapause survival of *A. fukutai* after being held at 15 °C and 12L:12D for one month and subsequently at 11 °C and 10L:14D for 6 months within parasitized *A. chinensis* eggs. Bars are mean + SE and different letters above the standard error bars indicate significant differences (Tukey’s HSD, *p* < 0.05).

**Figure 4:**
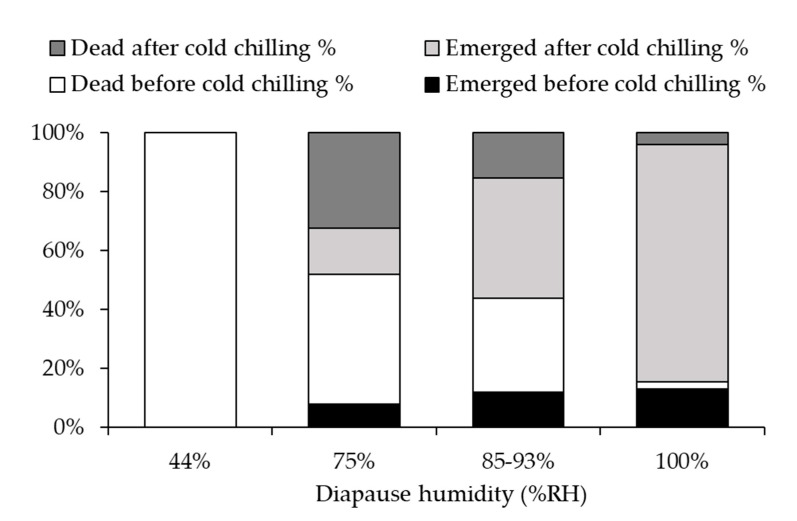
Survival and emergence of *A. fukutai* larvae pre- and post-cold chilling at different humidity conditions (see Table 1). Values are the percentage of emerged and dead individuals within the parasitized *A. chinensis* eggs.

Percent survival of diapausing *A. fukutai* individuals within parasitized hosts was significantly affected by humidity (*χ*^2^ = 46.1, df = 3, *p* < 0.001) but not by clutch size (*χ*^2^ < 0.1, df = 1, *p* = 0.999) or the interaction between these two factors (*χ*^2^ = 0.4, df = 3, *p* = 0.936) (Figure 3). No parasitoids survived at 44% RH, and all of them were dead at the larval stage before the onset of cold chilling (Figure 3). Survival rate increased with humidity, reaching 94.6% at 100% RH (Figure 3 and Figure 4). The percentages of female offspring (mean ± SE) were 87.8 ± 6.1%, 84.7 ± 5.0% and 73.8 ± 5.2% at 75%, 85–93% and 100% RH, respectively; it was not affected by humidity (*χ*^2^ = 1.1, df = 2, *p =* 0.562) or clutch size (*χ*^2^ = 0.1, df = 1, *p* = 0.710), and there was no significant interaction between these two factors (*χ*^2^ = 0.5, df = 2, *p* = 0.782). The post-diapause developmental time to adult emergence was 58.9 ± 1.1, 59.3 ± 0.8 and 50.6 ± 0.2 days at 75%, 85–93% and 100 %RH, respectively. There was no difference between the 75% and 85–93% RH treatments, but developmental time was shorter at 100% RH than the other treatments (*F*_2,364_ = 126.7, *p* < 0.001). Although the parasitized eggs used for 75% RH treatments were obtained from the first generation, while those used for 83–95% were obtained from the second generation, there was no difference in post-diapause developmental time between these two groups. Overall, about 10% of progeny of the first generation developed into adults without diapause at the most suitable humidity condition (100% RH) (Figure 4).

### 3.3. Optimal Temperature Regimes

**Figure 5:**
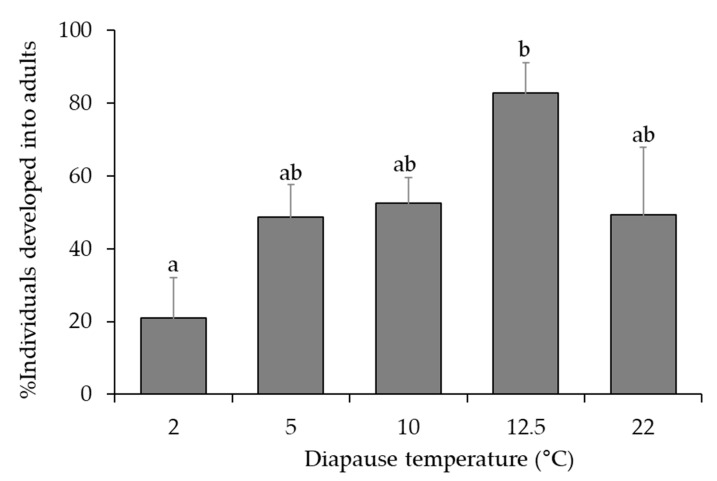
Effect of temperature regimes (see Table 1) on diapause survival of *A. fukutai* after being held for cold chilling in the dark and at 100% RH for 6–7 months or at normal rearing conditions continually (22 °C, 100% RH and 14L:10D). Bars are mean + SE and different letters above the standard error bars indicate significant differences (Tukey’s HSD, *p* < 0.05).

Percent survival of non-diapause *A. fukutai* individuals (second generation) within parasitized hosts under the normal room conditions (22 °C, 100% RH, 14L:10D) was 82.3 ± 3.8 (*n* = 59 parasitized host eggs). Percent survival of diapausing *A. fukutai* individuals within parasitized hosts was significantly affected by the temperature regimes during diapause (*χ*^2^ = 12.1, df = 4, *p* < 0.001), but not by the clutch size (*χ*^2^ = 2.5, df = 1, *p* = 0.113) or the interaction between these two factors (*χ*^2^ = 4.5, df = 4, *p* = 0.339); survival rate increased from 21.0% at 2 °C to 82.8% at 12.5 °C (Figure 5). When the parasitoid progeny were held under the normal room conditions (22 °C, 100% RH, 14L:10D) continually, adult wasps emerged from 55.0% of the parasitized hosts (i.e., developed into third generation) with a mean developmental time of 31.6 ± 0.1 days (*n* = 15) while the rest of them completed development in 269.0 ± 0.0 days (*n* =22). The realized clutch size was consistent across different temperature treatments (*F*_4,147_ = 2.0, *p* = 0.096) (Table 2) and was not significantly correlated with host egg size (*F*_1,116_ = 2.4, *p* = 0.124). The percentages of female offspring were not different among the different temperature treatments (*χ*^2^ = 0.7, df = 4, *p =* 0.954), and were not affected by the realized clutch size (*χ*^2^ = 0.2, df = 1, *p* = 0.681) or the interaction of these two factors (*χ*^2^ = 0.9, df = 2, *p* = 0.932) (Table 2). Post-diapause developmental time decreased with increased temperature (*F*_3,527_ = 360.0, *p* < 0.001) (Table 2). Post-diapause longevity of adult females emerged from 12.5 °C was longer than those from 2 °C (log-rank test, *χ*^2^ = 4.4, df = 1, *p* = 0.036), but the temperature regimes did not affect the lifetime number of hosts parasitized (*F*_1,44_ < 0.1, *p* = 0.834) (Table 2).

## 4. Discussion

We showed that adult *A. fukutai* emerged in June 2018 from parasitized *A. chinensis* eggs collected in May 2018 in northern Italy. In addition, adult wasps emerged in June and July from the outdoor trials under semi-natural conditions in southern France. From the field collections, we also had *A. chinensis* adults emerged in June 2018. This shows a good synchronization of adult emergence between the parasitoid and the beetle in northern Italy or southern France. Based on the laboratory rearing and bioassays, we found the majority of the progeny of the first (after diapause) generation entered diapause under normal room conditions with suitable temperatures and long-day photoperiods, but part of the progeny of the first generation or even second generation could develop into adults in the same year. This suggests an obligatory diapause for the majority of the first generation.

The diapause pattern of *A. fukutai* is generally similar to that of other host-specific egg parasitoids of woodboring beetles, such as *Oobius agrili* Zhang and Huang and *O. primorskyensis* Yao and Duan (Hymenoptera: Encyrtidae), which attack the emerald ash borer *Agrilus planipennis* F. (Coleoptera: Buprestidae) [5,38,39]. Although it is still unknown what mechanisms trigger the induction and termination of diapause, as well as the factors affecting the proportions of non-diapausing progeny of the first generation in *A. fukutai*, it is well-known that the physiological state of the female parent of other species prior to and at the time of oviposition can influence the proportion of her progeny entering diapause [2,7,38]. For example, the proportion of diapausing progeny increases with parental adult age in *O. primorskyensis* [37]. Thus, the life cycle of *A. fukutai* may be univoltine in colder temperate zones, but multivoltine in warmer temperate zones, such as in South China, where up to three generations were reported [30,31].

The long period of obligatory diapause, however, poses challenges in laboratory rearing of this parasitoid. Our first attempt to rear diapausing *A. fukutai* in naturally laid host eggs of *A. chinensis* on potted young plants outside of the quarantine facility of EBCL in southern France resulted in a high mortality, and only about 37% of parasitoid individuals successfully developed. Insect eggs cannot defend themselves against attack by natural enemies, and many adult insect females place their eggs inconspicuously into plant tissue [40]. In woodborer beetles, some species lay eggs into the bark crack, under the bark tissue or deeply into plant tissue to protect them [41]. Trees produce sap right after any physical damage including beetle oviposition, and egg chambers are often filled with sap, which could inhibit or kill the eggs or young larvae. For example, on some resistant poplar varieties, callous tissue overgrows the oviposition wounds quickly and can kill eggs of the Asian longhorned beetle *Anoplophora glabripennis* (Motschulsky) (Coleoptera: Cerambycidae) [42]. We observed in several cases that *A. chinensis* eggs or newly hatched larvae were drowned in or stuck to death by sap. Desurmont et al. [43] showed that plant tissue produced by several *Viburnum* species following oviposition by the invasive viburnum leaf beetle *Pyrrhalta viburni* can crush or expel the beetle eggs. Since parasitized host eggs would remain in the plant for up to 1 month (for non-diapausing generation) or 10 months (for the diapausing generation), they can be vulnerable to defensive tree responses. In this study, about 23% of parasitized eggs were found missing, expelled, or crushed due to the growth of wound tissue. In nature, adult *A. chinensis* females typically lay eggs in the lower trunk or exposed roots of trees close to the ground [12]. Although the natural mortality of diapausing *A. fukutai* larvae in the field is unknown, we suspect that the potted young trees that we used may have caused more mortality due to defensive tree responses. This mortality is one of the major reasons that we switched to rear diapausing *A. fukutai* in excised host eggs under the laboratory conditions.

For excised host eggs under the laboratory conditions, we found that low humidity was a main cause of mortality in both non-diapausing and diapausing *A. fukutai*. All *A. fukutai* were found dead at the larval stage at 44% RH due to desiccation even before the start of diapause treatment in mid-October (Figure 4). Survival rate increased with humidity, and was the highest at 100% RH. Tolerance to desiccation during diapause can be related to the habitats of the insects [2]. As pointed out previously, host eggs are normally embedded in the bark of a tree trunk near the ground, where the humidity may remain much higher than 44% RH due to the tree’s vascular system, the soil humidity, the presence of grass and leaves, as well as the shadow of the crown. The low resistance to desiccation of *A. fukutai* may reflect the natural host habitat that occurs primarily in the lower trunk of a tree, near the ground.

Cold tolerance is another important aspect of hibernal diapause of temperate insects [2,5,6]. We found that the survival rate of *A. fukutai* larvae was the lowest at 2 °C but similar across the other three temperature regimes, suggesting that the parasitoid can tolerate constant temperature as low as 2 °C, but it (or the tested population) is better adapted to warmer overwintering conditions. The northern Italy population of *A. fukutai* likely originated from Japan [27,32,44], although the exact location of its origin in Japan was unknown. This population seems to be adapted to subtropical regions based on its preferred winter diapausing temperatures. However, cold hardiness could vary among different geographical populations of the species [45,46]. We observed that post diapause developmental time of diapausing *A. fukutai* was inversely related to the diapause temperature. At the low temperature, the parasitoid emerged later (as their host would also likely emerge later) reflecting the adaption for a better phenological synchronization with the emergence of its host. Although we comparatively tested the reproductive fitness (longevity and fecundity) for the female wasps that emerged from only the lowest (2 °C) and highest (12.5 °C) chilling temperatures tested, life-time fecundity appears not to be significantly affected by the diapause temperature in this range. However, other factors such as the duration of cold chilling may also affect the diapause survival and post-diapause fitness [5]. In laboratory rearing, it seems to be ideal to terminate its diapause after a 6-month cold chilling during overwintering at these temperature regimes.

## 5. Conclusions

In summary, we have determined the optimal relative humidity (100% RH) and temperature (12.5 °C) conditions for diapause survival of *A. fukutai*. The most efficient method for the rearing of this parasitoid is to extract parasitized eggs and hold them for diapause at these conditions for 6 months. This information will facilitate maintenance of laboratory colonies as well as for conducting research and rearing of this parasitoid. As the only known specialist egg parasitoid of *A. chinensis*, *A. fukutai* has some important traits as a classical biological control agent (host specificity, female-biased sex ratio and being synchronized with the host). The parasitoid is still present even at low residual host populations due to extensive eradication efforts to remove *A. chinensis* in northern Italy, where the parasitoid could be mass-reared and released to increase its impact or help with the current eradication efforts, as it could be capable of locating hosts even at low host density, especially in remote areas. This parasitoid has also a great possibility to be accidently introduced or to be pre-approved for intentional introduction before an anticipated *A. chinensis* invasion in other regions (i.e., proactive biological control). Therefore, further studies should evaluate the potential of *A. fukutai* as a classical biological control agent for *A. chinensis,* and the current colony of the parasitoid may be preserved in insectaries for potential use in the future, in case the adventive population of the parasitoid in northern Italy will eventually become extinct after successful eradication of this invasive pest.

## Figures and Tables

**Table 1:** Different humidity conditions and temperature regimes tested for diapause survival of *A. fukutai* larvae within parasitized *A. chinensis* eggs.

**Humidity Conditions at 11 °C and 10L:14D ^1,2^**				
**Humidity (% RH)**	**Humidity Control**		**No. of Parasitized Host Eggs**	
44	Saturated K_2_CO_3_ solution		24	
75	Saturated NaCl solution		54	
85–93 ^3^	Saturated KCl or KNO_3_ solution		59	
100	Distilled water		72	
**Temperature Regimes at 100% RH and Dark ^1,2^**				
**First Temperature (°C)**	**Duration (month)**	**Second Temperature** **(°C)**	**Duration (month)**	**No. of Parasitized Host Eggs**
2	5	12.5	1	25
10	1	5	6	71
10	6	10	0	83
12.5	6	12.5	0	30
22	All time	22	All time	11

^1^ Parasitized host eggs were extracted from logs and surface-treated with 5% bleach solution prior to being individually placed in 24-well cell plate inside a humidity box for diapause development. ^2^ After a 6-month (humidity test) and 6- or 7-month (temperature test) chilling period, all parasitized host eggs were moved to 22 °C (14L:10D) until emergence of wasps. ^3^ Humidity was changed to 93% RH after one month at 85% RH.

**Table 2:** Effects of temperature regimes (see Table 1) on the developmental time and post-diapause reproductive fitness of *A. fukutai*.

Temperature Regime (°C)	Realized Clutch Size ^1,2^	% Female Offspring ^1,2^	Post-Diapause Developmental Time (days) ^1,2^	Longevity (Days) ^1,3^	Lifetime Fecundity ^1,3^
2	12.8 ± 1.0 a	89.0 ± 2.5 a	72.5 ± 1.9 a	9.6 ± 1.1 a	3.0 ± 0.5 a
5	10.7 ± 0.8 a	80.8 ± 3.0 a	71.8 ± 1.0 a		
10	10.0 ± 0.6 a	82.3 ± 2.6 a	51.5 ± 0.8 b		
12.5	11.6 ± 0.8 a	87.3 ± 2.5 a	34.9 ± 0.5 c	15.1 ± 2.2 b	3.1 ± 0.4 a
22	11.7 ± 0.5 a	85.4 ± 1.4 a			

^1^ Values are mean ± SE, and different letters within the column indicate significant difference (*p* < 0.05, ANOVA, Tukey HSD). The longevity was subject to Survival Analysis (log-rank test). ^2^ Data were pooled from all parasitized hosts within each treatment. ^3^ Longevity and lifetime fecundity (number of hosts parasitized) were tested only for adult females emerged from 2 and 12.5 °C treatments.

## Data Availability

Data are available upon request.
